# The Effect of *Griffonia simplicifolia* on Pain Intensity, Central and Peripheral Sensitization, and Pain Modulation in Healthy Volunteers—A Randomized, Double-Blinded, Placebo-Controlled Crossover Trial

**DOI:** 10.3390/nu18101609

**Published:** 2026-05-19

**Authors:** Anselm Johannes Schlemmer, Sascha Hammer, Simon Fandler-Höfler, Kordula Lang-Illievich, Helmar Bornemann-Cimenti

**Affiliations:** 1 Department of Radiology, Medical University of Graz, 8036 Graz, Austria; anselm.schlemmer@medunigraz.at; 2 Department of Anaesthesiology and Intensive Care Medicine, Medical University of Graz, 8036 Graz, Austria; sascha.hammer@medunigraz.at (S.H.); kordula.lang-illievich@medunigraz.at (K.L.-I.); 3 Department of Neurology, Medical University of Graz, 8036 Graz, Austria; simon.fandler@medunigraz.at; 4 Department of Anaesthesiology and Intensive Care Medicine, State Hospital Güssing, 7540 Güssing, Austria

**Keywords:** *Griffonia simplicifolia*, 5-hydroxytryptophan, 5-HTP, pain, central sensitization, peripheral sensitization, quantitative sensory testing, conditioned pain modulation, allodynia, hyperalgesia, wind-up

## Abstract

**Background**: The plant *Griffonia simplicifolia* is marketed as a dietary supplement; it is said to have antidepressant and sleep-promoting properties. Its main ingredient, 5-hydroxytryptophan (5-HTP), is the immediate precursor of serotonin and crosses the blood–brain barrier, thereby enhancing central serotonergic neurotransmission. Reduced serotonergic activity has been associated with affective disorders, sleep disturbances, and impaired central pain modulation. Despite this neurobiological rationale, evidence for analgesic efficacy remains limited. This study investigated the effects of *Griffonia simplicifolia* on peripheral and central sensitization and descending pain inhibition. **Methods**: In a randomized, double-blind, placebo-controlled crossover trial, 18 healthy volunteers underwent quantitative sensory testing (QST). Participants received 100 mg *Griffonia simplicifolia* orally once daily for 28 days or matching placebo. Sensory parameters were reassessed, followed by repetitive phasic heat application (RPHA) to induce short-term peripheral and central sensitization. After a 4-week washout period, participants crossed over to the alternate intervention. **Results**: A total of 17 participants completed the study. *Griffonia simplicifolia* showed no significant effect on acute pain perception after RPHA (β = −4.17; 95% CI −14.44 to 6.10; *p* = 0.401). The only significant difference was an increased distance of mechanical allodynia in the verum group (β = 0.82; 95% CI 0.05–1.59; *p* = 0.038). No differences were observed in thermal detection or pain thresholds, pressure pain thresholds, conditioned pain modulation, wind-up ratio, mechanical pain sensitivity, or flare area. Mild, transient adverse events occurred in two participants (11%) during *Griffonia simplicifolia* intake. **Conclusions**: *Griffonia simplicifolia* demonstrated limited effects on experimentally induced pain mechanisms compared with placebo and was well tolerated. Increased distance of allodynia may reflect serotonergic facilitation of pronociceptive pathways, suggesting an enhanced central and peripheral sensitization. Larger controlled trials are required to clarify its impact on pain perception.

## 1. Introduction

The seeds of *Griffonia simplicifolia*, an African leguminous plant, are a rich natural source of 5-hydroxytryptophan (5-HTP), an endogenous intermediate in the biosynthesis of serotonin. Structurally, 5-HTP is an aromatic amino acid derived from L-tryptophan via hydroxylation at the 5-position of the indole ring, leading to increased polarity while preserving its ability to cross the blood–brain barrier [1]. Once in the central nervous system, 5-HTP is rapidly decarboxylated by aromatic L-amino acid decarboxylase to serotonin (5-hydroxytryptamine, 5-HT).

Despite significant advances in perioperative and chronic pain management, pain remains a major clinical challenge [2]. It is associated with impaired quality of life, delayed recovery, increased healthcare utilization, and a higher risk of chronification and opioid consumption [2,3]. Insufficient efficacy and adverse effects, highlighting the need for novel and well-tolerated therapeutic approaches, often limit current analgesic therapies [4]. In this context, naturally derived compounds with potential modulatory effects on pain pathways have gained increasing scientific interest [5].

Reduced serotonin levels have been implicated in the pathophysiology of depression, anxiety disorders, and sleep disturbances [6]. In addition to central effects, serotonergic pathways play a key role in nociceptive processing, including modulation of descending inhibitory pain pathways and peripheral sensitization [7,8].

In humans, 5-HTP ‘s effects have been most explored in depressive disorders, where studies suggest possible antidepressant properties, although the overall quality of evidence remains limited by small sample sizes and methodological heterogeneity [9]. Beyond mood disorders, 5-HTP has been investigated for its potential role in reducing binge-eating behavior through enhanced satiety signaling and modulation of impulse control [10]. Effects on sleep appear to be mediated both by serotonergic mechanisms and downstream melatonin synthesis, with variable impacts on sleep architecture [11,12]. 5-HTP shows potential for reducing motion sickness in children, particularly when combined with magnesium [13].

Clinical evidence shows mixed results depending on chronic pain conditions. Randomized controlled trials reported significant improvements in patients with fibromyalgia, including reductions in pain intensity, fatigue, and sleep disturbances [14]. Similarly, double-blind studies in patients with chronic tension-type headache and migraine showed decreased headache frequency and reduced analgesic use following 5-HTP administration [15,16]. In migraine prophylaxis, clinical improvements have been reported, attributed to effects on serotonin turnover [17]. Other trials did not demonstrate clear superiority over placebo in primary headache disorders [18,19].

Collectively, these findings suggest a possible analgesic potential of serotonergic interventions involving 5-HTP. However, most available evidence has been derived from studies using isolated or purified 5-HTP rather than standardized *Griffonia simplicifolia* extracts. *Griffonia simplicifolia* represents a complex phytochemical matrix that may differ from purified 5-HTP with regard to composition, bioavailability, and pharmacodynamic properties. Findings obtained with isolated 5-HTP cannot be directly extrapolated to *Griffonia*-based preparations [20]. Consequently, controlled human studies specifically investigating the effects of *Griffonia simplicifolia* on pain processing and nociceptive modulation remain scarce. Experimental human pain models may therefore help to better characterize potential effects of *Griffonia simplicifolia* on peripheral and central pain mechanisms under standardized conditions.

Analgesic drug effects can be broadly attributed to three key mechanisms: peripheral sensitization, central sensitization, and pain modulation. Peripheral sensitization can be assessed using quantitative sensory testing such as thermal and mechanical detection thresholds, pressure pain thresholds, and vibration testing. Central sensitization is typically evaluated by dynamic paradigms, including mechanical allodynia and temporal summation (wind-up) induced by repetitive thermal or pinprick stimuli. Descending pain inhibition is examined in conditioned pain modulation (CPM) protocols. Clinically, acute pain is predominantly driven by peripheral sensitization at the site of injury, whereas chronic pain involves a combination of persistent peripheral and central sensitization mechanisms [21,22].

The primary aim of this study was to examine whether administration of *Griffonia simplicifolia* attenuates acute pain perception following repetitive phasic heat application (RPHA). Secondary, we investigated whether quantitative sensory testing (QST) can provide insight into possible effects of *Griffonia simplicifolia* on peripheral and central sensitization as well as on descending pain inhibition.

## 2. Materials and Methods

### 2.1. Trial Design and Participants

The investigation was conducted as a randomized, placebo-controlled crossover trial in healthy volunteers. The study was approved by the Ethics Committee of the Medical University of Graz (1308/2024, approved on 11 March 2025). The protocol was further registered at www.clinicaltrials.gov (NCT06893822, accessed on 17 June 2025).

Healthy adults (≥18 years) were recruited via public notice within the university setting. Exclusion criteria comprised hypersensitivity to *Griffonia simplicifolia*, as well as a history of current pregnancy or breastfeeding, neurological, dermatological, or cardiovascular disorders. Individuals with prior or current chronic pain conditions and those receiving ongoing analgesic therapy were excluded. The use of analgesics, including anticonvulsants, and antidepressants with analgesic indication, sleep medication, or St. John’s wort was prohibited throughout the study period.

Eligibility was assessed prior to enrollment. Potential participants received detailed oral and written information regarding study procedures and provided written informed consent. Participants were informed of their right to withdraw at any time without adverse consequences for their medical care.

### 2.2. Randomization and Blinding

The allocation sequence was created using an online randomization platform (https://www.randomizer.org/, accessed on 9 March 2025). Randomization was performed with equal allocation (1:1) to both groups. Labelling, allocation concealment, and blinding of the active compound and placebo were carried out by an independent staff member who had not been involved in study procedures. Both preparations were dispensed in indistinguishable boxes labelled with the study title, participant identification number, and the designation “Substance 1” or “Substance 2.” Participants as well as all study personnel were blinded to treatment allocation and sequence. Unblinding was performed after study completion by an independent staff member.

### 2.3. Interventions and Course of the Study

**Figure 1:**
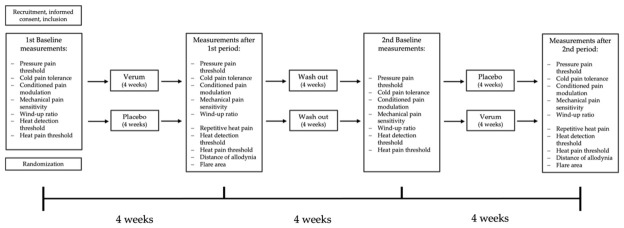
Schematic chart of the course of the study.

Each participant was supplied with 28 capsules containing either 100 mg of *Griffonia simplicifolia* (Nutricost, Vineyard, UT, USA) or a visually indistinguishable placebo manufactured in the pharmacy Casa Medica Dr. Peyer KG (Graz, Austria). Participants were instructed to administer one capsule every 24 h, with the final dose taken on the morning of the study visit. Adherence was supported by scheduled mobile phone reminders. At each visit, remaining capsules were counted and discarded. Following a 4-week washout period, participants received the alternate study medication in a second package containing 28 capsules. The course of the study is visualized in Figure 1.

Following enrollment, participants were randomized to one of two treatment sequences (100 mg *Griffonia simplicifolia* or placebo). Baseline assessments were obtained prior to treatment initiation.

At the initial visit, demographic data were collected using a standardized questionnaire. Pressure pain threshold (PPT), conditioned pain modulation (CPM), cold pain tolerance (CPT), mechanical pain sensitivity (MPS), wind-up-ratio (WUR), heat detection and pain thresholds (HDT, HPT) were assessed.

After 28 days of continuous intake of the assigned intervention, PPT, CPM, WUR, and MPS were reassessed.

**Figure 2:**
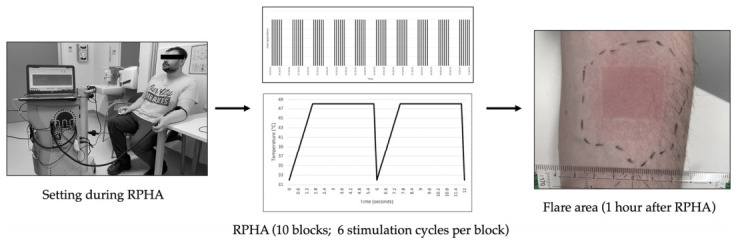
Repetitive phasic heat application (RPHA): Experimental setting.

Subsequently, repetitive phasic heat application (RPHA) was applied (Figure 2). A 3 × 3 cm area on the mid-volar aspect of the nondominant forearm was stimulated using a TSA-II NeuroSensory Analyzer (Medoc Ltd., Advanced Medical Systems, Ramat Yishai, Israel). The thermode temperature increased from a baseline of 32.0 °C to 48.0 °C at a rate of 10 °C/s, remained at peak temperature for 6 s, and then returned to 32.0 °C. This stimulation cycle was repeated six times per block, followed by a 30 s intermission. In total, 10 blocks with six repetitions each were conducted [23].

At the conclusion of the final stimulation block, perceived repetitive heat pain intensity was quantified using a numeric rating scale (NRS; 0–100). Assessments of HDT, HPT, distance of allodynia (DA), and the flare area (FA), were conducted 60 min after completion of the last heating sequence.

Following a 4-week washout phase, a new baseline assessment was obtained prior to crossover to the alternate intervention, which was administered for 28 days. Thereafter, the identical experimental procedures and outcome assessments were repeated.

### 2.4. Measurements

Repetitive heat pain intensity (RHP) after RPHA was defined as the primary outcome. The other QST parameters were defined as secondary outcomes.

#### 2.4.1. Repetitive Heat Pain

Immediately following the final thermal stimulation block of RPHA, repetitive heat pain intensity (RHP) was quantified using a numeric rating scale (NRS; 0–100).

#### 2.4.2. Pressure Pain Threshold and Conditioned Pain Modulation

CPM reflects endogenous inhibitory pain control based on the “pain inhibits pain” principle [24]. The test stimulus consisted of pressure delivered by a Wagner Pain Test Model FPK algometer (Greenwich, CT, USA) to the adductor pollicis brevis muscle of the nondominant hand (probe area: 1 cm^2^). Pressure was increased at a rate of 0.5 kg/cm^2^/s until the participant first reported pain (PPT1; kg/cm^2^). Subsequently, participants put the dominant hand and wrist in 4 °C water until reaching 40 points on a 0–100 NRS, defining cold pain tolerance (CPT) in seconds. Immediately thereafter, PPT was reassessed at the identical site (PPT2). The CPM effect was calculated as the relative change in PPT (kg/cm^2^): [(PPT2 − PPT1)/PPT1 × 100]. A positive value indicates activation of endogenous inhibitory pathways [25].

#### 2.4.3. Mechanical Pain Sensitivity and Wind-Up Ratio

Temporal summation was evaluated by comparing pain intensity of a single pinprick stimulus with that evoked by a series of 10 stimuli (256 mN, 1 Hz). Stimuli were applied within a 1 cm^2^ area located on the volar forearm of the nondominant arm. Pain intensity at the end of each train was rated on a 0–100 NRS.

The response to the single pinprick stimulus represented mechanical pain sensitivity (MPS). The wind-up ratio (WUR) was defined as the mean pain rating of three stimulus trains divided by the mean rating of single stimuli [26].

#### 2.4.4. Heat Detection and Pain Thresholds

Heat detection threshold (HDT; °C) and heat pain threshold (HPT; °C) were determined at all four study visits. After each intake period HDT and HPT were assessed after RPHA in accordance with the protocol of the German Research Network on Neuropathic Pain [22]. A TSA-II NeuroSensory Analyzer (Medoc Ltd., Advanced Medical Systems, Ramat Yishai, Israel) was used at the 3 × 3 cm thermal stimulation site.

Participants terminated the thermal ramp via a stop button upon first perceiving warmth (HDT) or when the sensation became painful (HPT; described as pulling, burning, or stinging). Each parameter was assessed three times, and mean values were calculated.

#### 2.4.5. Distance of Allodynia and Flare Area

Mechanical allodynia was mapped using a 128 mN von Frey filament 60 min after thermal stimulation. Starting at the center of the thermal stimulation area, stimuli were applied along four radial axes in 0.5 cm/s steps, first moving centripetally and subsequently centrifugally. The distance at which a sharp unpleasant sensation emerged or disappeared was recorded. Eight measurements were averaged to determine the distance of mechanical allodynia (DA; cm).

The size of the flare area (FA; cm^2^) was marked with a water-soluble pen (Figure 2). The size of the flare area was calculated using the public domain software ImageJ version 1.54g (National Institutes of Health, Bethesda, MD, USA).

#### 2.4.6. Adverse Events

At each visit, participants were systematically queried regarding potential adverse effects.

### 2.5. Sample Size Calculation and Statistical Analysis

The study was designed as a two-period, two-treatment crossover trial. Sample size estimation was based on data from Jürgens et al., who applied RPHA in healthy volunteers [24]. The reported mean maximal pain was 72.2 on a 0–100 numerical rating scale, with a standard deviation (SD) of 5.4. To adopt a conservative approach, the SD was doubled to 10.8.

A similar study by Lang-Illievich et al. investigating the effects of palmitoylethanolamide in healthy volunteers used this approach, enrolling 14 participants (power 0.8, alpha 0.05, dropout 20%) and successfully demonstrating significant treatment effects [27]. Under these assumptions, a total of 18 participants would provide 88% power to detect a mean difference of 10 units on the pain scale using a paired-samples *t*-test with a two-sided alpha of 0.05, accounting for an anticipated dropout rate of 20%.

Analyses were performed using IBM SPSS Statistics version 29.0 (IBM, Armonk, NY, USA). Descriptive statistics were calculated after data acquisition. Treatment effects were analyzed using linear mixed-effects models with treatment, period, and sequence as fixed factors and subject as a random effect. Baseline values were included as covariates where applicable. For outcomes without baseline assessment, post-treatment values were analysed within the same modelling structure. Carryover effects were explored indirectly via the sequence effect. In the absence of a significant sequence effect, treatment effects were interpreted as unbiased. Period effects were examined to detect potential time- or learning-related influences. Results are reported as adjusted mean differences (β estimates) with 95% confidence intervals. Statistical inference was based on model-derived *t* tests. A two-sided *p* value < 0.05 was considered statistically significant.

## 3. Results

From September to December 2025, 18 participants (5 women and 13 men) were included. A total of 17 participants completed the study without deviating from the protocol and were included into the statistical analysis. The participants’ characteristics are summarized in Table 1.

### 3.1. Baseline Data

No statistically significant sequence effect could be detected (Table 2). Only PPT1 (β = 1.42; 95% CI −0.19 to 3.02; *p* = 0.082) and PPT2 (β = 1.34; 95% CI −0.19 to 2.88; *p* = 0.082) showed a trend toward a sequence effect, suggesting a minor, non-significant influence of treatment order on the outcome (β = 1.34; 95% CI −0.19 to 2.88; *p* = 0.082).

Period effects were generally non-significant. Only HDT showed a small, isolated period effect without concomitant treatment differences (β = −0.63; 95% CI −1.24 to −0.02; *p* = 0.045). These findings indicate that measurements were stable across study periods and that the washout was effective.

### 3.2. Effect of Griffonia simplicifolia on QST Parameters

*Griffonia simplicifolia* showed no significant effect on acute pain perception after RPHA (RHP, primary outcome) (β = −4.17; 95% CI −14.44 to 6.10; *p* = 0.401).

When considering that the secondary outcomes treatment effects were largely absent, with exception of distance of allodynia (DA), which exhibited a statistically significant difference favoring the verum (β = 0.82; 95% CI 0.05–1.59; *p* = 0.038). PPT, CPT, CPM, MPS, WUR, HDT, HPT, and FA did not show any significant treatment effects. The results are shown in Table 3.

### 3.3. Dropouts and Adverse Effects

One participant dropped out for reasons unrelated to the study shortly after the 1st study visit. No participants discontinued the study due to adverse effects, and no serious adverse events were observed. Two participants reported dyspeptic symptoms when the study medication was taken on an empty stomach during administration of the active compound.

## 4. Discussion

The results of this study could not demonstrate a significant analgesic effect of *Griffonia simplicifolia* (or the main content 5-HTP) compared to placebo following repetitive phasic heat application. The serotonergic system generally plays a complex and bidirectional role in pain processing. 5-HTP, as a serotonin precursor, increases central and peripheral serotonin levels, which can have both pro-nociceptive and anti-nociceptive effects depending on receptor subtype, tissue context, and pain modality. Centrally, increased serotonin can enhance descending inhibitory pathways, leading to antinociception via activation of spinal 5-HT receptors, particularly 5-HT1A, 5-HT2A, 5-HT2C, and 5-HT3 subtypes, as demonstrated in animal models and supported by human studies using serotonin depletion paradigms [8,28,29]. Conversely, pro-nociceptive effects are prominent peripherally, where serotonin sensitizes primary afferent nociceptors and can lead to hyperalgesia and allodynia, especially in inflammatory and neuropathic pain states [8,28]. The duality is further complicated by neuroplastic changes in chronic pain, where the same receptor subtype (e.g., 5-HT7) may exert opposite effects depending on the presence of nerve injury or sensitization [30,31,32]. The effect of 5-HTP administration on peripheral and central serotonin levels leads to increased nociceptive processing and expansion of allodynic areas, particularly via 5-HT3 receptor activation at the spinal level [30,33,34]. Furthermore, 5-HTP administration increases plasma beta-endorphin levels, suggesting involvement of endogenous opioid systems, though the relationship between these elevated opioid levels and actual pain perception appears complex [35]. Randomized controlled trials in humans confirm that manipulation of serotonin levels (via precursors or depletion) alters pain thresholds and tolerance, with effects varying by pain modality and receptor distribution [28,36]. The anti-inflammatory and analgesic effects of 5-HTP have been demonstrated in preclinical models, where it inhibits COX-2 and iNOS expression through modulation of the MAPK-ERK pathway, reducing inflammatory mediators like nitric oxide and IL-6 [37].

Another objective of the study was to provide insights into the underlying mechanism by which *Griffonia simplicifolia* or 5-HTP affects pain perception, using quantitative sensory testing. The controlled heat stimulus used in this experimental setup was used to induce a short-term central and peripheral sensitization. This effect was utilized to investigate the influence of the test substance on the underlying pain mechanisms [23].

Our study demonstrated an increased distance of mechanical allodynia one hour after application of the heat stimulus in the verum group. Since multiple secondary QST endpoints were evaluated without formal correction for multiple testing, this isolated significant finding should be interpreted with caution and regarded as exploratory rather than confirmatory.

Allodynia is an increased sensitivity to pain triggered by normally painless stimuli. It develops through peripheral sensitization, central sensitization, and an imbalance between excitation and inhibition in the pain pathways [38,39]. The mechanisms by which 5-HTP appears to promote allodynia seem to involve both central and peripheral sensitization. Following conversion to serotonin, descending facilitatory pathways in the spinal cord are activated. In rats 5-HTP induces bladder hypersensitivity by activating spinal 5-HT3 receptors, as the pro-nociceptive effect was attenuated by intrathecal ondansetron, a 5-HT3 antagonist [35]. Spinal serotonin acting on 5-HT4, 5-HT6, and 5-HT7 receptors promotes the development and maintenance of secondary allodynia and hyperalgesia [40]. At the peripheral level, primary afferent nociceptors are sensitized by serotonin through 5-HT2B and 5-HT2C receptors (sensitizing TRPV1 channels and activating calcium-activated chloride channels) and 5-HT4, 5-HT6, and 5-HT7 receptors (augmenting primary afferent input promoting central sensitization) [41,42,43]. Clinically, oral 5-HTP (100 mg) induced rectal allodynia in healthy controls and rectal hyperalgesia in hypersensitive IBS (irritable bowel syndrome) patients, accompanied by increased plasma levels of the main serotonin metabolite 5-hydroxyindoleacetic acid (5-HIAA) [44].

Serotonergic pathways exert a complex, bidirectional influence on nociceptive processing, with distinct peripheral and central mechanisms [8]. In the central nervous system, serotonin contributes to analgesia primarily via descending inhibitory pathways from the brainstem to the spinal cord, where it can suppress pain transmission [45]. In contrast, at the peripheral level serotonin may facilitate nociception by sensitizing primary afferent neurons and promoting pronociceptive signaling through specific receptor subtypes [7]. Accordingly, modulation of serotonin metabolism via 5-HTP may result in either analgesic or pronociceptive effects depending on the anatomical site and receptor profile involved.

Furthermore, the present study demonstrated that a 4-week washout period interval appears sufficient to prevent relevant carryover effects in a crossover design investigating *Griffonia simplicifolia*. This may be useful for future crossover studies. The study by Santucci et al. reported a sequence effect with a 2-week washout period [18].

In this study, two participants (11%) experienced mild and transient side effects during intake of the verum. No serious side effects were observed. These results support the assumption that *Griffonia simplicifolia* is a well-tolerated substance This is consistent with other randomized, double-blind, placebo-controlled studies comparing 5-HTP with a placebo [14,15,16,18,19].

Several limitations should be acknowledged. First, although no statistically significant difference between *Griffonia simplicifolia* and placebo was observed for the primary endpoint repetitive heat pain (RHP), these findings should be interpreted with caution, because of the small sample size. The wide confidence intervals despite prior sample size calculation suggest limited statistical power and indicate considerable uncertainty around the estimated treatment effect. Therefore, the absence of statistical significance does not exclude the possibility of clinically relevant analgesic effects, which should be further investigated in adequately powered trials.

A further limitation is the imbalance in sex distribution, with a predominance of male participants. Sex-related differences in pain perception, serotonergic signaling, and analgesic response have been described previously and may influence the generalizability of the findings [46,47]. Therefore, potential sex-specific effects of *Griffonia simplicifolia* could not be adequately assessed in the current trial and should be addressed in future studies with larger and more balanced cohorts.

Second, the present study was designed to evaluate the effects of *Griffonia simplicifolia* under realistic supplementation conditions using a fixed-dose regimen over a four-week intervention period. However, this design does not permit conclusions regarding potential dose–response relationships, optimal dosing strategies, or the underlying biological mechanisms. In addition, no direct biochemical assessments (e.g., serotonergic biomarkers or plasma levels) were performed to confirm biological activity or treatment adherence.

Most evidence regarding serotonergic modulation and pain processing is based on isolated 5-HTP supplementation rather than standardized *Griffonia simplicifolia* extracts, limiting direct comparability with the present study.

Third, quantitative sensory testing depends on active participant cooperation and subjective reporting. Pain perception is subjective and may be influenced by day-to-day fluctuations in mood, stress, sleep quality, and overall physical condition. Furthermore, 5-HTP was administered as a single substance and not in combination with adjunctive compounds (e.g., magnesium) [13,48]. Finally, the inclusion of healthy volunteers limits the transferability of the results to clinical pain populations.

Future research should aim to validate these findings in larger, adequately powered cohorts to improve statistical robustness and enable subgroup analyses. In addition, biomarker-based approaches could be considered, such as the measurement of plasma or urinary serotonin and its metabolite 5-hydroxyindoleacetic acid (5-HIAA), which have been shown to increase following 5-HTP administration [20,44,49]. Co-administration with vitamin B6 (pyridoxine), a cofactor of aromatic L-amino acid decarboxylase, may enhance the peripheral conversion of 5-HTP to serotonin and thereby influence bioavailability [48].

## 5. Conclusions

In this randomized, double-blind, placebo-controlled crossover study, 4-week *Griffonia simplicifolia* supplementation demonstrated limited effects on experimentally induced pain in healthy volunteers. Although a significantly increased distance of mechanical allodynia was observed, this finding should be interpreted cautiously, as multiple secondary endpoints were assessed without correction for multiple comparisons. Consequently, the observed effect should be considered exploratory and hypothesis-generating rather than confirmatory. Nevertheless, the findings may indicate a potential influence of *Griffonia simplicifolia* on mechanisms related to central and peripheral sensitization, highlighting the complex role of serotonergic pathways in pain modulation. Future adequately powered clinical trials are warranted to further investigate the underlying mechanisms and potential clinical relevance.

## Figures and Tables

**Table 1:** Participants’ characteristics.

Characteristics	Enrolled (*n* = 18)	Completed (*n* = 17)
Sex (f/m)	5/13	4/13
Age (years)	37.27 ± 17.75	36.45 ± 12.66
Weight (kg)	79.39 ± 18.12	80.41 ± 18.13
Height (cm)	176.5 ± 8.28	177.24 ± 7.92
BMI (kg/m^2^)	25.38 ± 4.85	25.52 ± 4.96
Compliance of verum intake (%)	---	99.74
Compliance of placebo intake (%)	---	98.74

**Table 2:** Baseline Data.

			Sequence Effects	
Parameter	Baseline 1	Baseline 2	β (95% CI)	*p*
PPT1 (kg/cm^2^)	3.59 ± 1.12	4.02 ± 2.22	1.42 (−0.19 to 3.02)	0.082
CPT (s)	80 ± 63.30	82.47 ± 82.5	53.98 (−14.68 to 122.65)	0.115
PPT2 (kg/cm^2^)	4.22 ± 1.43	4.33 ± 2.24	1.34 (−0.19 to 2.88)	0.082
CPM (%)	18.44 ± 15.66	9.56 ± 16.77	0.86 (−12.51 to 14.23)	0.893
MPS (NRS 0–100)	8.9 ± 6.21	8.15 ± 10.58	−0.60 (−6.52 to 5.32)	0.832
WUR	3.86 ± 3.66	3.12 ± 1.49	−1.09 (−2.58 to 0.40)	0.141
HDT (°C)	34.22 ± 0.7	34.7 ± 2.49	0.16 (−0.64 to 0.96)	0.672
HPT (°C)	43.44 ± 3.37	45.55 ± 2.19	0.58 (−1.61 to 2.77)	0.579
HPT-HDT (°C)	9.21 ± 3.09	10.86 ± 2.95	0.42 (−1.63 to 2.47)	0.669

**Table 3:** Results.

			Treatment Effects	
Parameter	Verum	Placebo	β (95% CI)	*p*
PPT1 (kg/cm^2^)	4.32 ± 1.79	4.49 ± 1.91	−0.22 (−1.63 to 1.19)	0.742
CPT (s)	94.91 ± 78.07	86.12 ± 82.03	7.16 (−37.16 to 51.49)	0.735
PPT2 (kg/cm^2^)	4.56 ± 2.28	4.59 ± 1.79	−0.07 (−1.53 to 1.36)	0.923
CPM (%)	4.61 ± 16.05	7.08 ± 14.1	−2.11 (−11.44 to 7.23)	0.638
MPS (NRS 0–100)	7.77 ± 8.95	7.22 ± 4.47	0.73 (−4.07 to 5.54)	0.750
WUR	3.24 ± 2.43	3.35 ± 1.6	−0.16 (−1.67 to 1.51)	0.824
HDT (°C)	35.54 ± 0.84	35.5 ± 1.08	−0.004 (−0.61 to 0.61)	0.989
HPT (°C)	39.47 ± 2.21	39.91 ± 2.31	−0.47 (−1.58 to 0.64)	0.382
RHP (NRS 0–100)	59.71 ± 20.25	63.38 ± 19.04	−4.17 (−14.44 to 6.10)	0.401
FA (cm^2^)	40.9 ± 10.76	37.65 ± 8.09	3.02 (−3.01 to 9.04)	0.303
DA (cm)	4.71 ± 1.41	3.88 ± 1.02	0.82 (0.05 to 1.59)	0.038 *

* *p* < 0.05.

## Data Availability

Data are available on request from the corresponding author.

## Abbreviations

The following abbreviations are used in this manuscript:

5-HIAA	5-hydroxyindoleacetic acid
5-HT	5-hydroxytryptamine
5-HTP	5-hydroxytryptophan
CPT	Cold pain tolerance
CPM	Conditioned pain modulation
DA	Distance of Allodynia
FA	Flare area
HDT	Heat detection threshold
HPT	Heat pain threshold
MPS	Mechanical pain sensitivity
PPT	Pressure pain threshold
RHP	Repetitive heat pain
RPHA	Repetitive phasic heat application
QST	Quantitative sensory testing
WUR	Wind-up ratio
